# Accuracy and responses of genomic selection on key traits in apple breeding

**DOI:** 10.1038/hortres.2015.60

**Published:** 2015-12-23

**Authors:** Hélène Muranty, Michela Troggio, Inès Ben Sadok, Mehdi Al Rifaï, Annemarie Auwerkerken, Elisa Banchi, Riccardo Velasco, Piergiorgio Stevanato, W Eric van de Weg, Mario Di Guardo, Satish Kumar, François Laurens, Marco C A M Bink

**Affiliations:** 1 Institut de Recherche en Horticulture et Semences UMR1345, INRA, SFR 4207 QUASAV, F-49071 Beaucouze, France; 2 Research and Innovation Center, Fondazione Edmund Mach, San Michele all’Adige, Trento, Italy; 3 Better3Fruit, Rillaar, Belgium; 4 DAFNAE, Dipartimento di Agronomia Animali Alimenti Risorse Naturali e Ambiente, viale Università 16, 35020 Legnaro (PD), Università, degli Studi di Padova, Italy; 5 Wageningen UR Plant Breeding, Wageningen University and Research Center, Wageningen, The Netherlands; 6 The New Zealand Institute for Plant & Food Research Limited, Private Bag 1401, Havelock North 4157, New Zealand; 7 Biometris, Wageningen University and Research Center, Wageningen, The Netherlands

## Abstract

The application of genomic selection in fruit tree crops is expected to enhance breeding efficiency by increasing prediction accuracy, increasing selection intensity and decreasing generation interval. The objectives of this study were to assess the accuracy of prediction and selection response in commercial apple breeding programmes for key traits. The training population comprised 977 individuals derived from 20 pedigreed full-sib families. Historic phenotypic data were available on 10 traits related to productivity and fruit external appearance and genotypic data for 7829 SNPs obtained with an Illumina 20K SNP array. From these data, a genome-wide prediction model was built and subsequently used to calculate genomic breeding values of five application full-sib families. The application families had genotypes at 364 SNPs from a dedicated 512 SNP array, and these genotypic data were extended to the high-density level by imputation. These five families were phenotyped for 1 year and their phenotypes were compared to the predicted breeding values. Accuracy of genomic prediction across the 10 traits reached a maximum value of 0.5 and had a median value of 0.19. The accuracies were strongly affected by the phenotypic distribution and heritability of traits. In the largest family, significant selection response was observed for traits with high heritability and symmetric phenotypic distribution. Traits that showed non-significant response often had reduced and skewed phenotypic variation or low heritability. Among the five application families the accuracies were uncorrelated to the degree of relatedness to the training population. The results underline the potential of genomic prediction to accelerate breeding progress in outbred fruit tree crops that still need to overcome long generation intervals and extensive phenotyping costs.

## Introduction

Developing new fruit tree cultivars is a time-consuming process for two main reasons. First, the long juvenile phase delays the acquisition of phenotypic data that are necessary to identify genotypes that will produce marketable fruits satisfying both farmer and consumer demands.^1^ Second, breeding programmes are often organized in two or more successive steps. The initial *large diversity* step involves phenotyping a large number of candidates over two or three years at a single location. Those candidates presenting favourable characters are identified and enter the *cultivar evaluation* step comprising extensive phenotypic evaluation over a longer period at multiple contrasting locations. The early identification of promising genotypes at the *large diversity* step would enable fruit tree breeders to enhance genetic gain per year and react more efficiently to changing demands by reducing breeding cycle length.^2,3^

Molecular markers have been developed to help fruit tree breeders for early identification of interesting genotypes. Indeed, associations between markers and genes underlying agronomic traits are the basis for marker-assisted selection (MAS)^4^ which can be performed on young seedlings as well as for selecting well-combining parents. Similar to other crops,^5^ MAS has been applied to apple breeding schemes manipulating few major genes including resistance genes for scab, fire blight, mildew, aphids and genes involved in fruit firmness or storability (see e.g. Baumgartner *et al.*^6^). However, traditional MAS is ineffective when many genes of small effects are segregating, and reliable markers have not been identified. In this context, Meuwissen *et al*.^7^ proposed to skip the quantitative trait locus (QTL) detection step in favour of using all available markers in a genome-wide prediction approach termed genomic selection. In genomic selection, a training population on which both phenotypic and genotypic data are available is used to construct a prediction model which is subsequently applied to estimate genomic breeding values (GBVs) of individuals that only have genotypic data. Genomic selection often targets additive genetic variation, i.e. estimation of breeding values, but may also account for dominance or higher-order genetic variances in order to estimate genotypic values. Genomic selection could complement MAS for polygenic traits and thus obviate the need of phenotyping at the *large diversity* step. Genomic selection would strongly enhance breeding efficiency by decreasing generation interval and increasing the accuracy of breeding value estimates and selection intensity.

Most genomic selection studies in plants have been conducted on annual crops such as maize,^8–13^ barley^14^ and wheat,^15^ where inbred lines are the main focus of selection. A few studies on perennial outbreeding plants have been published, particularly on forest trees,^16–19^ switchgrass,^20^ oil palm,^21^ Japanese pear^22^ and apple.^23^ These studies used simulations and/or cross-validation, in a defined set of plant material genotyped at high density and phenotyped in a common set of environments, to evaluate accuracy of genomic selection. An exception is the work of Asoro *et al.*^24^ who compared the results of genomic selection to phenotypic selection and MAS after two selection cycles for the improvement of β-glucan concentration in oats. Based on random cross validation on seven full-sib (FS) families, Kumar *et al*.^23^ reported high accuracies of genomic selection, between 0.67 and 0.89, for six fruit quality traits in apple, and concluded that genomic selection is a credible alternative to conventional selection for these traits. Applicability and success of genomic selection to other traits and to designs commonly used by commercial fruit breeders have not yet been reported.

The purpose of this study was to assess the prospects of genomic selection in current apple breeding populations for 10 *culling* traits that are related to productivity and fruit external appearance. Individuals that do not perform well for culling traits are eliminated prior to any harvest. Genomic selection could provide accurate estimated breeding values of these traits so that breeders could skip phenotypic evaluation. This is the first study in fruit crops where GBVs are calculated for individuals that do not belong to the training population but to additional material developed and studied by breeders. Accuracy of genomic prediction was estimated by comparing predicted GBV and phenotypic data of application individuals. Realized selection differential was assessed on the basis of phenotypic differences between individuals with the highest and lowest GBV in a large full-sib family. The influence of factors such as trait heritability and relatedness between application and training populations on accuracy of genomic prediction was evaluated. The composition of the training population in relation to accuracy of prediction and impact of genomic prediction of culling traits on improving breeding scheme efficiency were discussed.

## Material and methods

### Plant material

The training population, developed in the EU-funded HiDRAS project^25^ for Pedigree Based QTL analysis,^26^ consisted of 20 full-sib (FS) families with a total of 977 individuals. These FS families were obtained from breeding programmes from four research institutes (INRA, France; JKI, Germany; UNIBO and LFW, Italy) at the start of the HiDRAS project (see e.g. Kouassi *et al*.^27^ for more details) and resulted from crosses among 24 parents (Supplementary Table S1) which were related to each other via common ancestors. The parents, intermediate ancestors and founders (individuals with unknown ancestors) were included in pedigree data.

The application population consisted of five FS families developed within two European apple breeding programmes, Novadi and Better3Fruit, located in France and Belgium respectively. A total of 1390 individuals from these five families were used in this study, the family size ranged from 109 to 662 (Table 1). The application families resulted from crosses between nine parents where one parent (313) was involved in two crosses (Table 1). Out of the nine parents, five were also the parents of progenies in the training population (Supplementary Table S1). The pedigree relationships among the parents and FS families of the training and application populations were abundant and were visualized with Pedimap software^28^ as shown in Supplementary Fig. S1.

### Trait phenotypes

Phenotypic data for the training population were available from the HiDRAS project and were partly described by Kouassi *et al*.^27^ The individuals of the training population were each evaluated at one location managed by the involved research institutes, and data were collected during at least two seasons over a period of three years, from 2003 to 2005. The individuals were not replicated. Our study focused on culling traits, which are scored before or at harvest. Productivity-related traits namely pre-harvest dropping and fruit cropping were assessed along with external appearance traits such as fruit size, per cent of russet, fruit cracking, attractiveness and four components of skin colour, ground colour, over-colour, per cent over-colour and type of colour. All traits were visually scored on an ordinal scale (1–5, Supplementary Table S2). Twenty-nine reference genotypes (Supplementary Data 1, not part of training population) were scored in all locations and were used to adjust phenotypic data for location and year effects (similar to Bink *et al*.^29^). As in Bink *et al*.,^29^ the best linear unbiased predictions (BLUPs) of genotypic effects of the individuals in the training population were used as phenotypes for the development of genome-wide prediction model.

For the application population, the same traits as in the training population were scored at harvest, except that skin colour was assessed as attractiveness of colour and not as the four component traits scored in the training population. Phenotypic data for the application population were collected, without replication, in 2013 at two sites, i.e. two families in Seiches (FR) and three families in Rillaar (BE), all managed by the involved apple breeders of Novadi and Better3Fruit, respectively.

### Marker genotypes

The genotypic data of the training population and their progenitors were obtained from a FruitBreedomics experiment on Pedigree Based Analyses by using the Illumina 20K SNP array^30^. This same experiment provided SNP data on two additional FS families, ‘Telamon’ × ‘Braeburn’ (162 individuals) and ‘Jonathan’ × ‘Prima’ (25 individuals), that were used to improve genotype imputation (see below and Supplementary Table S1). These two latter families did not have phenotypes for the studied traits. This experiment also provided genetic linkage maps, which included a total of 15.8K mapped SNP markers,^30^ of the training and additional families. Subsequently, a set of 7651 SNP markers passed the filtering criteria on absence of null-alleles, disturbing additional SNP at the probe set, and genotyping interference from paralogous loci, thus showing robust performance across this germplasm (Van de Weg & Di Guardo, personal communication). The across-families integrated map of these 7651 SNP markers was used here (version of July 4 2014).

Twenty-four individuals from the five application families were genotyped with the 20K SNP array following the standard Illumina protocol detailed in the study of Chagné *et al*.^31^ to check parent–offspring consistency and to help phasing the markers in the imputation step (see below).

All 1390 individuals of the five application families were genotyped with an array of 512 SNPs using the QuantStudio 12K Flex Real-time PCR system and OpenArray technology (Life Technologies, Carlsbad, CA, USA). These 512 SNPs were a subset of the 15.8 K mapped SNPs. Details on SNP selection process are given in Supplementary Data 2, and the positions of the selected SNPs on the genetic map are shown in Supplementary Fig. S2. Samples consisting in 10 ng DNA were mixed with 2.5 μl of TaqMan OpenArray Genotyping Master Mix (Life Technologies, Carlsbad, CA, USA) in a 384-well plate. Samples were subsequently loaded onto the OpenArray plate using the QuantStudio 12K Flex OpenArray AccuFill System. Following PCR, allelic discrimination results were analysed using the TaqMan Genotyper software v. 1.2 (Life Technologies, Carlsbad, CA, USA).

### Genotypic data curation

The TaqMan OpenArray (512 SNPs) genotypic data resulted in 364 SNPs that could be reliably scored in the application population. These 364 SNPs were checked for Mendelian segregation errors and frequency of observed recombination events using the FlexQTL software^29^ (www.flexqtl.nl). The additional curation on the 15.8K SNPs (see previous paragraph) introduced a substantial mismatch between the 364 SNPs scored with the TaqMan array and the 7651 robust SNPs, i.e. 178 out of the 364 SNPs were not considered as robust. The genotypic data on these 178 “non-robust” SNPs for the training population were added to those on the 7651 robust SNPs to enhance the accuracy of imputation in the application population. Positions of the robust and non-robust SNPs scored with the TaqMan array are shown in Supplementary Fig. S2.

The resulting matrix of genotypic data comprised 7829 SNPs on 2661 individuals. The 1271 individuals genotyped with the 20K array had sporadically missing data, and 1390 individuals from the application FS families had substantially (>95%) missing data.

### SNP genotype imputation

The imputation of genotypic scores for missing SNP data was done in two steps by using AlphaImpute software,^32^ which uses pedigree, linkage and linkage disequilibrium information. In both steps, default values were used for all software parameters except for the windows sizes to account for the number of SNPs in the data set (values for *CoreAndTailLengths* and *CoreLengths* ranged between 100 and 300 SNPs, and between 50 and 200 SNPs, respectively). In the first step, AlphaImpute was applied to the families of the training population, their progenitors, the two additional FS families and the 24 individuals of the application FS families, all genotyped with the 20K array. In the second step, AlphaImpute was applied to the 1390 individuals of the application families utilizing the completed data from the first step as reference.

### Variance components and heritability

To estimate the heritability of the 10 traits, the phenotypic data were first adjusted for fixed effects, i.e. year and location effects using all available data. Then a linear mixed model (Supplementary Data 3) was used to estimate the additive ($\sigma_{\text{a}}^{2}$) and residual ($\sigma_{\text{e}}^{2}$) variance components using only individuals of the training population. Narrow-sense heritability for each of the 10 traits was estimated as $h^{2}=\frac{\sigma_{\text{a}}^{2}}{\sigma_{\text{a}}^{2}+\sigma_{\text{e}}^{2}}$ Estimates of variance components were obtained with the R package *breedR*.^33,34^

The pedigree-based relationship matrix was obtained with the R package *pedigree*^35^ and the mean pedigree-based relatedness between each application family and the training population was calculated.

### Genomic relatedness

Genomic relatedness between application population and training population were computed using the imputed genotypic data. The matrix of genotypic data (**X**) with individuals of the training and application population in columns and SNPs in rows was first standardized using means and standard deviations of genotypic data computed for each SNP in the training population. Then the genomic relationship matrix (**G**) was computed as

$$\text{G}=\frac{\text{W}\prime \text{W}}{p}$$

where **W** is the standardized version of the matrix **X**, and *p* is the number of SNPs.^36^

Because of the standardization, the mean of the elements of **G** pertaining to the pairwise relatedness of an individual of the application population to all individuals of the training population was expected to be zero.^37^ Following Clark *et al*.,^38^ three fractions of the training population were considered to summarize the relatedness of application individuals and families to the training population. The *top 10 relatedness* of each application individual was calculated as the mean of the 10 highest values among the elements of **G** corresponding to the relatedness of an application individual to the individuals of the training population. Likewise, the top 5% and 25% relatedness of each application individual to the training population were calculated. Subsequently, the top 10, top 5% and top 25% relatedness of each application family to the training population was calculated as the mean of these variables within each family.

### Genomic prediction

The BayesCπ method,^39^ as implemented in GS3 software (Legarra *et al*., 2011, http://snp.toulouse.inra.fr/˜alegarra), was used to estimate GBVs. In this method, the parameter π can be interpreted as the proportion of SNPs that truly affect the trait. Likewise the distribution of estimated SNP effects may reveal information on the genetic architecture of the trait. Once the prediction model (Supplementary Data 3) was established based on the training population, the GBVs in the application population, $\hat{g}$, were estimated.

### Accuracy of predictions and realized selection differential

The accuracy of genomic predictions was calculated as the correlation between the GBV $\hat{g}$ and the phenotypic scores in the application population. Pearson correlations were used for all traits and Spearman rank correlations for traits with highly skewed distributions. Accuracy was calculated separately within the five application families due to confounding with locations. As shown in the Appendix, the accuracy calculated in this way is expected to be proportional to the square root of narrow sense heritability. The correlations were calculated with the function cor.test in R software,^34^ which also provided estimates for the asymptotic confidence intervals (based on Fisher’s z-transformation) for Pearson correlations.

The realized selection differential within each application family was estimated as the difference in mean phenotypic scores between the individuals with the highest GBV and the individuals with the lowest GBV. The significance of these differentials was assessed via a Student’s *t-*test. We selected 50 individuals from both tails of the distribution of GBVs, which equated to selected fractions of 7.5% in case of the largest application family, AF1-Da66, which comprised 662 individuals (Table 1). A directional realized selection differential was also estimated as the difference in mean phenotype of the individuals with the most favourable GBV and all individuals within each application family.

Note that the trait colour was scored in the application families as attractiveness of colour, while it was scored as four components, i.e. ground colour, over-colour, per cent over-colour and type of colour in the training population. We calculated accuracy of colour predictions using the four components correlated to the same phenotypic scores (on attractiveness of colour). Likewise, we calculated realized selection differential as the difference in mean phenotypic scores for attractiveness of colour between the selected extreme individuals for GBV calculated for the four components of colour.

## Results

### Distribution of trait phenotypes

**Figure 1:**
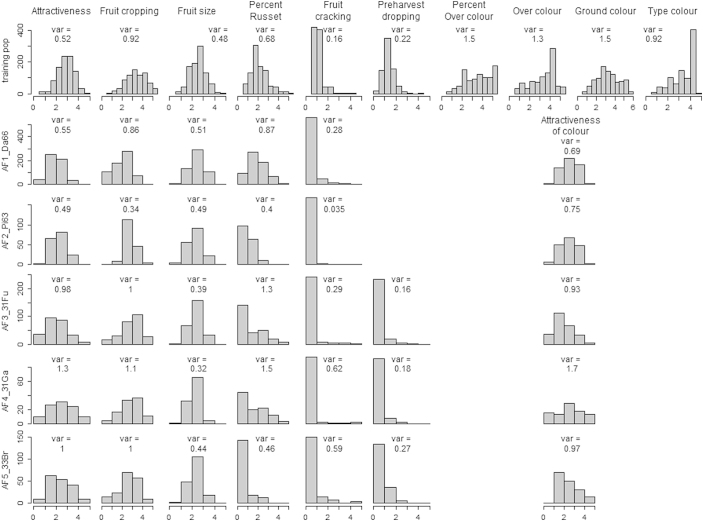
Within-training population distribution of genotypic BLUP (upper row) and within-family distribution of phenotypic data (five lower rows) for traits scored at harvest. Variances are indicated. Non-plotted distributions correspond either to a trait not scored in a family (pre-harvest dropping in AF1_Da66 and AF2_Pi63 families) or, for colour, to components not scored in application families.

In the training population the distributions of phenotypes (= genotypic BLUP values) varied greatly among the 10 traits considered in this study (Figure 1). The skewness was high and negative for fruit cracking, moderate and negative for per cent of russet and pre-harvest dropping and moderate and positive for type of colour and over-colour. The distributions of the other traits were almost symmetrical. These differences between traits were also present in the distributions of residuals in the quantile–quantile plots (Supplementary Fig. S3). Due to the pre-adjustment for year and site effects, the range of phenotypes was slightly increased, [0–6] for all traits. However, for fruit cracking, the highest phenotypic value was just above 4, which exemplified the highly negatively skewed distribution.

The distributions of phenotypes varied greatly among traits and between the five application families (Figure 1). Highly asymmetric distributions were observed for fruit cracking, pre-harvest dropping and per cent of russet (except the AF1_Da66 family) and showed the highest frequency in the first, highly desired, class. The distributions for the other traits were almost symmetric, but with narrow ranges as the extreme scores (1 and 5) were hardly present for fruit size in all families and for attractiveness of colour in AF1_Da66 and AF2_Pi63 families. The phenotypic variances in the application population varied greatly over families and over traits, from 0.035 for fruit cracking in AF2_Pi63 family to 1.7 for colour in AF4_31Ga family.

### Distributions of SNP effects

**Figure 2:**
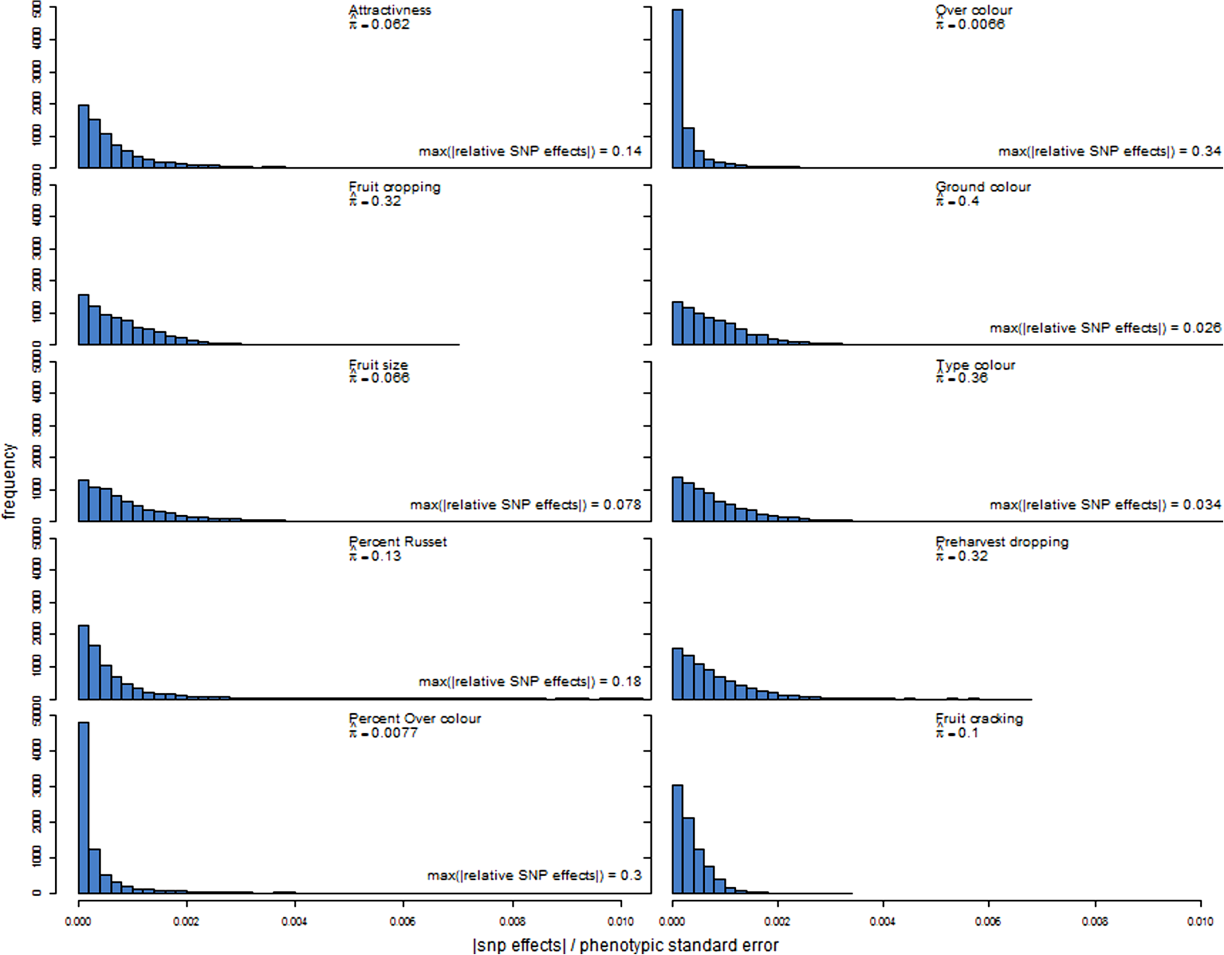
Distributions of absolute values of SNP effects relative to phenotypic standard deviation in the training population. Plots were truncated at 0.01 on the x-axis, and the highest absolute value of relative SNP effects for the trait is indicated when truncated. Variable $\hat{\pi }$ is the proportion of SNPs included in the genomic prediction model.

The distributions of estimated SNP effects in the prediction model varied greatly among traits (Figure 2). More than half of the SNP effects were very close to zero (i.e. less than 10^−4^) for per cent over-colour and over-colour. The distributions of SNP effects were more dispersed for the other traits. The relative SNP effects extended to very large values for per cent over-colour and over-colour, i.e. larger than 0.3, and also quite large values for attractiveness and per cent of russet, i.e. larger than 0.1, whereas the range of relative SNP effects for fruit cracking was very narrow, extending only to 0.0034 (Figure 2). The estimated probability (π) of marker inclusion in the prediction model varied between 0.007 (over-colour) and 0.397 (ground colour). Furthermore, between 3.7% (fruit size) and 7.0% (fruit cracking) of the markers included in the prediction models were actually genotyped in the application families rather than imputed.

### Accuracy of genomic prediction

Accuracy of predicting phenotypic scores was very low or negative when distributions of traits were very narrow or when these were highly skewed in the training or application populations. This was most apparent for fruit cracking and pre-harvest dropping in all families and per cent of russet in AF5_33Br family (Table 1). For these traits, the Spearman and Pearson correlations were in the same ranges (not shown). Accuracies ranged from 0.02 to 0.38 when distributions on traits were almost symmetrical, setting apart the colour components (Table 1). The low accuracies for fruit cropping correspond to a flat distribution of relative SNP effects which extended to a small value (less than 0.0069, Figure 2). By contrast, the higher accuracies for attractiveness, fruit size and per cent of russet correspond to distributions where some of the relative SNP effects were above 0.078 (Figure 2). The accuracies were close to 0 or negative for ground colour and type of colour and were moderate or high for over-colour and per cent over-colour, e.g. 0.50 for per cent over-colour in AF3_31Fu family (Table 1). These results suggest that attractiveness of colour is more strongly associated with over-colour and per cent over-colour than with the other two colour traits. Also, the moderate to high accuracies for over-colour and per cent over-colour corresponded to flat distributions of relative SNP effects with a large proportion of effects close to 0 and some extending up to 0.3 or higher. Comparison of the mean accuracy across all traits versus the four traits with almost symmetric phenotypic distribution (see Figure 1) indicate the strong influence of the phenotypic distribution of ordinal traits in the application families on accuracy of prediction. The 95% confidence intervals on the correlations were shorter for the larger families, i.e. varying from 0.16 in AF1_Da66 family (*n* = 662) to 0.37 in AF4_31Ga family (*n* = 109) (Table 1). The lengths of the confidence intervals were rather constant within families (not shown).

### Accuracy and heritability

**Figure 3:**
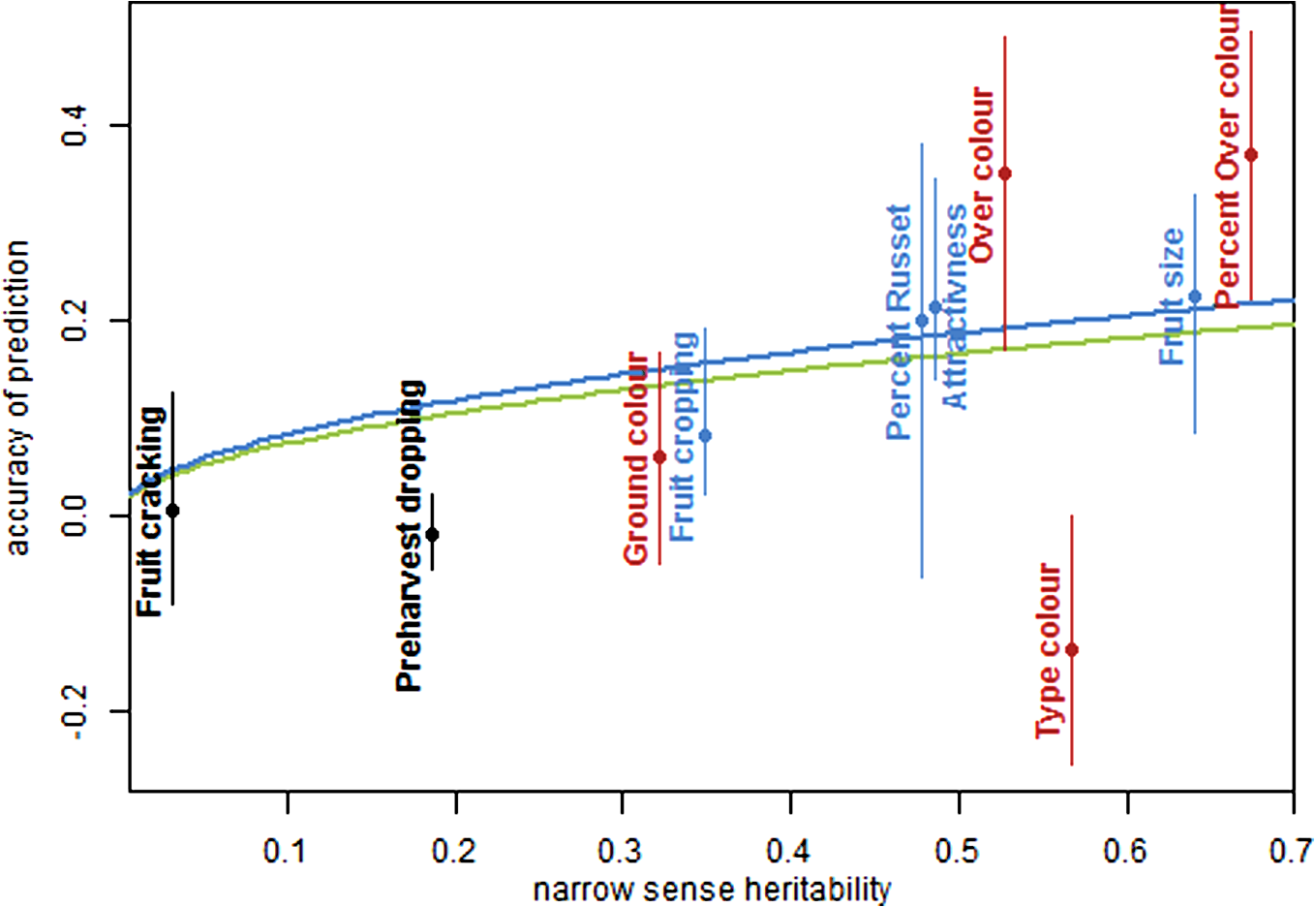
Effect of narrow sense heritability on accuracy of prediction. Points and vertical lines represent the mean and range in accuracy over families, respectively. In blue, the four symmetrically distributed traits (attractiveness, fruit cropping, fruit size and per cent of russet), in black, the two highly skewed traits (fruit cracking and pre-harvest dropping) and in red the four colour components compared to attractiveness of colour. The blue and green lines represent linear regressions without intercept of mean accuracy as a function of square root of heritability on the four symmetrically distributed traits and all traits, respectively.

In the training population, the narrow sense heritability of traits varied from 0.03 to 0.67 for fruit cracking and per cent over-colour, respectively. As expected, there was a clear positive trend between heritability and prediction accuracy, which was significant (*P* <1%) either considering all traits or considering the four symmetrically distributed traits (Figure 3). Obviously, this trend is mostly due to the fact that we calculated accuracy of predicting phenotypic scores and not genotypic values (see Appendix). Note that estimation of the heritability in the application population would have been more appropriate, but it was less meaningful here because data of only two or three un-replicated families were available per location.

### Genomic relatedness between application and training populations

Top 10, top 5% and top 25% relatedness between the application families and the training population varied in quite narrow ranges (Table 2). The ranking of the families was the same for the top 10 and top 5% relatedness and different from the ranking for top 25% relatedness and pedigree-based relatedness, which were also different from each other. The standard deviations of these relatedness within application families increased from top 25% to top 10 relatedness and was often close to 10% of the mean (Table 2). At the individual level, genomic relatedness between individuals of the application population and individuals of the training population varied between −0.31 and 0.59. The highest genomic relatedness were observed between individuals that shared a parent and thus individuals from one application family often had their most closely related individuals in one or a few training families. The levels of highest genomic relatedness for individuals of the AF5_33Br family were lower than those for individuals of the other families (refer to Supplementary Fig. S4).

### Accuracy and genomic relatedness

The relationship between accuracy of prediction and genomic or pedigree-based relatedness varied over traits and over measures of relatedness (Table 3). There was no association for most traits except a significant association for per cent of russet and mean top 25% relatedness. For attractiveness, fruit cropping, per cent of russet and over-colour, the correlation increased when increasing the number of highest values considered to calculate the mean relatedness, e.g. from 0.55 for top 10 relatedness to 0.68 for top 25% relatedness for attractiveness.

### Realized selection differential

**Figure 4:**
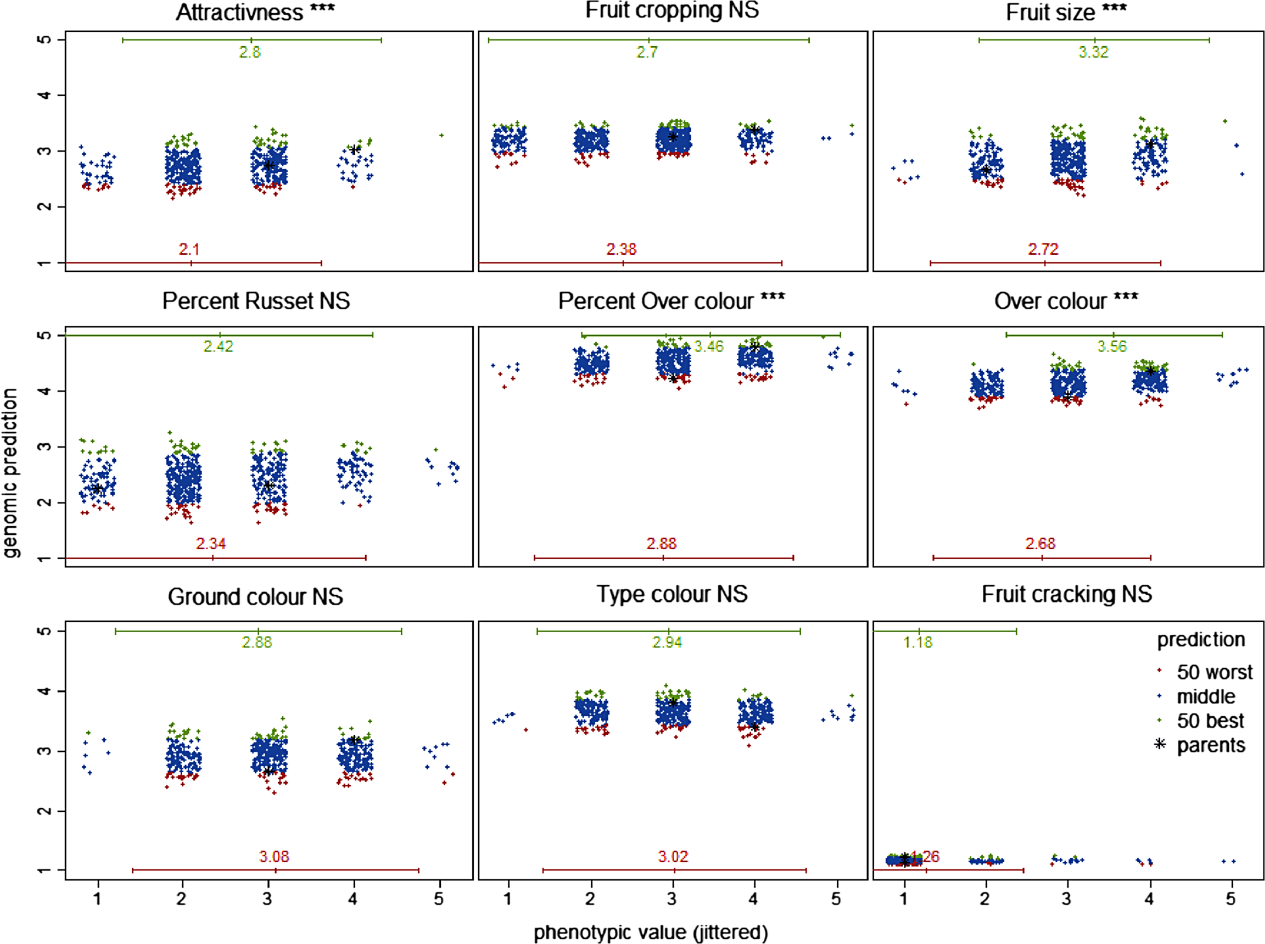
Relationship between phenotypic scores and predicted genomic breeding values in the AF1_Da66 family. The 50 (best) individuals with the highest predicted GBV are represented by green points, the 50 (worst) individuals with the lowest predicted GBV by red points, the other individuals by blue points. The black stars represent the parents. The horizontal green and red lines represent the interval of ±2 standard error around the mean of the groups of 50 individuals with the highest or lowest predicted GBV.

The realized selection differential in the (large) AF1_Da66 family was between 0.6 and 0.9 and highly significant for four traits (i.e. attractiveness, fruit size, over-colour and per cent over-colour, Figure 4). Conversely, it was almost absent, i.e. between −0.1 and 0.3, for the other traits (Figure 4, Supplementary Table S3). The directional realized selection differential was significant for the same traits in the AF1_Da66 family (Supplementary Table S4). The traits with significant responses also had the highest accuracy estimates, ranging between 0.21 and 0.34 in AF1_Da66 family (Table 1). The significant response for attractiveness for example implies that out of the 50 individuals with the highest GBV, none had the lowest phenotypic score of 1. Likewise, only one of the 50 individuals with the lowest GBV received a score of 4, and none the highest score of 5 (Figure 4). Similar trends were observed for fruit size, over-colour and per cent over-colour, but for these traits, very few individuals received the extreme scores of 1 or 5. By contrast, the responses for per cent of russet and fruit cropping were not significant and individuals with the highest and the lowest GBV received the lowest score of 1. Most application individuals with the extreme phenotypic scores for these four traits were in the middle of the distributions of GBV, i.e. neither belonged to the group of 50 individuals with the highest GBV nor to the group of 50 individuals with the lowest GBV.

In the other four application families, the realized selection differential was always significant for per cent over-colour, for three of them for attractiveness, per cent of russet and over-colour, for two of them for fruit size and for only one for fruit cropping (Supplementary Table S3). The results were slightly different for the directional realized selection differential, which was always significant for per cent over-colour, for three of them for per cent of russet, for two of them for fruit cracking and for one of them for attractiveness, fruit cropping, fruit size and over-colour (Supplementary Table S4). The realized selection differential was significant for six traits in AF4_31Ga (i.e. attractiveness, fruit cropping, fruit size, per cent of russet, over-colour and per cent over-colour, Supplementary Table S3), whereas it was significant only for three traits in AF2_Pi63 (i.e. attractiveness, per cent of russet and per cent over-colour, Supplementary Table S3). The largest realized selection differentials were observed in family AF4_31Ga for over-colour (2.38) and per cent over-colour (2.63).

## Discussion

This study reports encouraging results on genomic selection for traits that are scored before or at harvest in two European apple breeding programmes. Accuracy of genomic prediction of phenotypic scores varied with traits and families. Heritability was clearly a factor affecting accuracy in this study, whereas the effect of genomic relatedness between application and training population on accuracy was not significant. The realized selection differential in the largest FS family, AF1_Da66, was highly significant for four traits (attractiveness, fruit size, over-colour and per cent over-colour) and negligible for the other five traits.

### Factors affecting genomic prediction accuracy

#### Relatedness between training and application population

The relatedness between training population and application individuals is a key factor affecting prediction accuracy.^40,41^ Our training population was expected to be well-suited for genomic prediction in the application FS families as for each family one or both parents were also parents in the training population, except for AF5_33Br family (Supplementary Table S1, Supplementary Fig. S1). The dense and irregular structure of pedigree relationships between application and training FS families is a plausible explanation why none of the three measures of genomic relatedness provided a regularly spaced sample of relatedness. The later would have been more useful to test the relationship between relatedness and accuracy of genomic prediction. Nevertheless, the three measures of genomic relatedness did not address the same level of relationship. In the application FS families that shared a parent with the training FS families, the 10 most closely related individuals were indeed predominantly present in the training FS families with the shared parents. For individuals from the AF5_33Br family, the 10 most closely related individuals were distributed over 15 of the 20 training FS families (with lower levels of relatedness), but in more than 140 (out of 178) cases most of them were from ‘Pinova’ × ‘Reanda’ and ‘Rewena’ × ‘Pirol’ families, which share recent common ancestors with parent 338 (‘Priam’ × ‘Reka’). Thus the top 10 relatedness seemed mostly influenced by recent common ancestors. On the contrary, the top 25% most closely related individuals were distributed among 16–20 training FS families suggesting that top 25% relatedness was mostly influenced by more distant common ancestors.

The AF5_33Br family was the least related application family, but it did not consistently show the lowest accuracy for all traits. For example, accuracy of genomic prediction in AF5_33Br family was higher than in AF2_Pi63 family for fruit size, per cent over-colour and over-colour (Table 1). The absence of a clear trend between genomic relatedness and prediction accuracy could also be due to the rather large uncertainty of the estimated accuracies (Figure 3). Despite the large family sizes in the application population, the confidence interval lengths ranged between 0.16 and 0.37. Such large sampling errors of accuracy estimates were also observed by Wolc *et al*.^42^

These results emphasize the importance of the composition of the training population as all QTLs that are segregating in selection candidates of the application populations should also be present at reasonable allele frequency in the training population. The application of genomic selection in a single large bi-parental plant population, phenotyping only a subset, yield high accuracy on unphenotyped full-sibs.^43^ In such an application, the generation interval cannot be reduced while this is critical in perennial fruit crops. Thus, a more powerful training population in fruit crops should capture a large and genetically diverse collection of small bi-parental populations to maximize the relatedness of any selection candidate with multiple members of the training population.^41^ A larger diversity of the training population and the larger distance between training and application populations will require a higher marker density than for genomic selection in a single bi-parental population.

The advantage of using a multi-parental population for training, compared to training and application within a single bi-parental population, is to share the costs of genotyping and phenotyping the training population over a larger number of selection decisions. For fruit trees, this is particularly important as phenotyping costs are high due to the large space needed to grow trees and the long time required to evaluate traits of interest because of the juvenile period and because of the perennial nature of the crops.

#### Genetic architecture of the trait

The genetic architecture of the trait, which can be partly described by the number of QTLs and the distribution of their effects, is another key factor affecting accuracy of genome-wide predictions. This architecture is fixed for a given population—it may change by altering the composition of the population. We used the BayesCπ model because of its robustness to a large range of trait genetic architecture in terms of number of QTLs. Although the focus here was not on model inference, the number of QTLs (i.e. non-zero marker effects) influencing a trait may be postulated from the estimated proportion of SNPs (π) from the BayesCπ model. Indeed, Habier *et al.*^39^ showed in simulations that estimates of π reflected well the genetic architecture of the trait. For example, the estimated π of 0.007, 0.062 and 0.319 for over-colour, attractiveness and fruit cropping, respectively, would correspond to 55, 470 and 2500 QTLs, respectively. These estimated numbers of QTLs are orders of magnitude larger than what reported in previous QTL mapping studies,^29,44–53^ which is most likely due to the significance threshold used in QTL mapping studies but omitted in genomic prediction. Low π estimates, and SNPs of large effects (Figure 2), were generally observed for traits with moderate to high accuracies. For traits where the largest SNP effects were small, the size of the training population could have been not large enough to properly estimate SNP effects.

The realized selection differential, in the large family AF1_Da66, was significant for four traits (attractiveness, fruit size, over-colour and per cent over-colour). However, most individuals with extreme phenotypic scores for these traits were in the middle of the distributions of GBV, and thus would not have been identified for selection or culling purposes. This shows that the tails of the distributions for these traits were not well predicted, even if accuracies were high. Note that phenotypes were taken as indicators for the individuals’ true genotypic values and these phenotypes may have been imprecise for (some) individuals. In addition, the presence of non-additive genetic effects was ignored in the (additive) prediction model. Further exploration of genomic prediction models including dominance and epistasis would be appropriate as fruit tree crops are often vegetative propagated.^54^

#### Marker density and linkage disequilibrium in training population

The main hypothesis of genomic selection is that all QTLs will be in LD with at least one marker.^55^ The marker density in this study (six markers/cM) may be too low to have markers in strong/complete LD with each QTL and consequently the effect for such QTL is diffused over multiple SNPs, thereby increasing the earlier mentioned estimates of π for various traits. The diffusion of QTL effect over multiple (bi-allelic) SNPs may also occur when the QTL is multi-allelic.^56^ On the other hand, long stretches of LD might be present in the training population that comprised 20 FS families with moderate to large sizes and many recent common ancestors. The number of recombination events was consequently much lower compared to a population of unrelated individuals. The average *r*^2^ between the adjacent markers was 0.3, and the average *r*^2^ between markers separated by 0.2 cM, 2 cM (around 1 Mb) and 20 cM was 0.26, 0.17 and 0.045, respectively (Supplementary Fig. S5), which were almost identical to those reported by Kumar *et al.*^23^

#### Optimization of experimental setup

Using deterministic approaches and simplifying assumptions, several formulae have been proposed to predict the accuracy of genomic selection prior to any experiment.^57–59^ The reliable estimation of the “number of effective segments in the genome” as a function of genome size and effective population size was recently questioned.^60^ Notwithstanding, all formulae consistently identified heritability as a key factor affecting accuracy of genome-wide predictions, and this was confirmed by our results (Figure 3). The heritability in these formulae pertains to the additive genetic part of the precision of phenotypic data, and this narrow-sense heritability can be very high (≥0.95) when data are obtained from progeny testing.^60^ Likewise, the heritability of traits in apple and most fruit trees can be increased by averaging phenotypes from clonal replications or from multiple years when trees are not clonally replicated (the latter ignoring permanent environment effects). In all formulae to predict the accuracy of genomic selection, the size of the training population and heritability are mostly used together in a product. Consequently, when establishing a training population, economic parameters, such as costs of plantation, maintenance and phenotyping, must be considered to optimise the size of the training population and the number of replications that affect heritability and eventually maximize accuracy. Another way of raising heritability of traits could be the use of more objective assessment methods instead of visual scoring, for example using digital imaging for traits pertaining to fruit external appearance.

#### Phenotypic distributions

All traits were measured on an ordinal 1 to 5 scale but treated as continuous variables in our analysis. As the phenotypic data used for the training population were averaged over multiple years and also adjusted for year and site effects, we could consider them as continuous variables and we verified that the distributions of the residual terms in the training population were normal for all traits except pre-harvest dropping and fruit cracking (Supplementary Fig. S3). A more general approach for prediction is the use of an ordinal probit or threshold model^22^ that includes fixed effects (e.g. year and location effects and their interaction) influencing the raw phenotypic data. The threshold model holds a continuous latent variable underlying the observed ordinal scores, and this latent variable is described with fixed effects and genetic marker effects. In our case, however, fitting such models for estimation of these fixed effects was not possible because the application FS families were phenotyped in locations and years that were different from those of the training population. Montesinos-López *et al*.^61^ reported that ordinality of the phenotypic data is not problematic when the number of classes of an ordinal trait is large, i.e. not less than five, and the data approximated a normal distribution. Wang *et al*.^62^ extended the BayesCπ method to fit a threshold model for ordinal traits and reported very similar accuracies for the normal and threshold models for simulated traits with four or eight classes and an approximately normal distribution. The threshold model did yield superior accuracies for traits with four classes and highly asymmetric distributions.^63^

In our study, several traits, i.e. pre-harvest dropping, fruit cracking and per cent of russet in application family AF5_33Br, showed a very limited phenotypic variation with very skewed distributions (Figure 1), but even for these traits, distribution of genotypic BLUP in the training population was moderately asymmetric. Such asymmetric phenotypic distributions are frequently observed for these traits (Laurens, personal communication). This may be due to experimental conditions that do not favour expression of the defects to evaluate. These distributions gave rise to the very low accuracies obtained for fruit cracking (from −0.09 to 0.13, Table 1), for per cent of russet in AF5_33Br family (accuracy −0.06) or for pre-harvest dropping (accuracies between −0.06 and 0.02). Fruit cracking also had a very low heritability (0.03, Figure 3), lower than the value of 0.22 observed by Durel *et al.*,^64^ an intermediate estimated value for π in the BayesCπ model (0.1, Figure 2), and a short range of relative SNP effects, extending to less than 0.0034, all contributing to very low accuracies. To increase accuracy for this trait, one could consider a presence/absence classification of the defect and fit a binomial model as shown for root vigour in sugar beet by Biscarini *et al.*^65^ However, this approach ignores the intensity of the defect, when present. For binomial traits, Wang *et al.*^62^ showed that the accuracies obtained with the threshold BayesCπ method decreased when heritability and/or incidence were lower. Consequently, the low incidence, and heritability, of fruit cracking would probably yield low accuracies when applying the threshold version of the BayesCπ method.

Accuracies of genomic prediction for fruit cropping were also very low, except in AF4_31Ga (Table 1), while phenotypic variance was large for this trait and phenotypic distributions were almost symmetric in all application FS families, except in AF2_Pi63. Fruit cropping is often affected by biennial bearing and breeders will usually consider multiple years of phenotyping. Consequently, additional phenotypic data on the application population are needed before drawing reliable conclusions on genomic selection for fruit cropping.

#### Genotype by environment interaction

The application FS families and training population were phenotyped in different locations and years. Putative genotype by environment interactions due to differences in years and locations, were not considered and might have affected the prediction accuracy. Only parents were planted in the same plots as the application families, so there was an insufficient number of reference genotypes available to estimate genotype by environment interaction. However, as the training population was evaluated over three years and several locations, the SNP effects estimated to build the genomic prediction model reflect mean effects over years and locations, which would make predictions more robust to genotype × environment interaction. Additional phenotyping of the application populations is in progress, and using the average over multiple years may yield more stable estimates of the phenotypic performance (as a proxy for true breeding values), which could further increase the accuracy of the predicted breeding values. To study genotype by environment interactions in the context of genomic selection in perennial fruit crops, a collaborative initiative is underway to establish replicates of large reference populations in apple and peach at multiple sites throughout Europe.

#### Imputation of marker genotypes

Imputation of marker genotypes was seen as a tool to make genomic selection cost effective^66^ by genotyping selection candidates with a panel of evenly spaced low-density SNPs instead of the high-density panel used in the training population. Results from a simulation study revealed that the loss of accuracy using a low-density panel with markers every 10 cM was limited in a dairy-cattle like population.^66^ Likewise, the application of three levels of reduced density SNP panels (approximately one marker every 5, 0.7 and 0.35 cM) in pigs showed that imputation accuracy would be higher than 0.9 provided that both parents of individuals genotyped at low density are genotyped at high density.^67^ In our study, the mean interval length between the 364 usable markers was 3.7 cM with 29 intervals being larger than 10 cM and one interval exceeding 20 cM (Supplementary Fig. S2). For these regions the accuracy of imputation might have been reduced. As only 3.7% to 7.0% of the markers with the highest effects were actually genotyped in the application families, reduced imputation accuracy would probably result in a loss of prediction accuracy. Putative confounding factors hampered the assessment of the impact of imputation on prediction accuracy. The accuracy of imputation will be assessed in a forthcoming study.

### Comparison to previous studies

The only previous study concerning genomic selection on apple^23^ indicated higher accuracies, ranging between 0.67 and 0.89, for six fruit quality traits. Several factors may explain the discrepancy between the results of our study and those of Kumar *et al*.^23^ Kumar *et al.*^23^ estimated accuracies by cross-validation within a population of seven FS families obtained in a factorial mating design with four female and two male parents, and sampling for cross-validation was performed without taking into account family structure so that each individual in the validation set had full-sibs in the training set. Such a within FS family prediction is expected to result in high accuracies.^41^ In the present study, four application FS families shared one or both parents with some of the training FS families. In the work of Kumar *et al.*,^23^ the genotypic data were obtained with the 8K SNP array^31^ for all individuals under study, so imputation was used only for imputing sporadic missing data and not for a high amount of genotypic data as done in the present study. Based on the imputed datasets, the marker density was lower in Kumar *et al.,*^23^ however, the levels of LD were similar to those observed in the present study (Supplementary Fig. S5). In the study of Kumar *et al*.,^23^ the narrow-sense heritability of the traits studied varied between 0.19 and 0.60, thus there was no trait with very low heritability like fruit-cracking in the present study. Finally, in the work of Kumar *et al.*,^23^ the phenotypic data in the validation sets used to estimate accuracy of genomic prediction were obtained in the same orchard and with a common adjustment as phenotypic data in the training sets, thus avoiding potential genotype × environment interaction that would reduce accuracies.

### Optimization of apple breeding programmes

#### Genetic bases of apple breeding programmes

The training population represented the major founders of European and worldwide breeding programmes, in order of representation: ‘Golden Delicious’, ‘Delicious’, ‘McIntosh’, F2-26829-2-2, ‘Jonathan’, ‘Cox’ and some representation of ‘Common Antonovka’.^68^ Indeed, in terms of accuracy of genomic prediction or realized selection differential, no major differences were present between the five application FS families derived from breeding programmes in France (AF1_Da66, AF2_Pi63) and Belgium (AF3_31Fu, AF4_31Ga, AF5_33Br). The part of breeding programmes devoted to introgression of new resistance genes,^69^ as well as families descending from cultivar ‘Braeburn’ (that was absent in HiDRAS), could require a more diverse training population. However, as the current training population contained the major founders of the European breeding programmes, genomic prediction seems applicable for many crosses and juvenile FS families in ongoing breeding programmes, allowing selection prior to field-phenotyping. Finally, the static training population can evolve into a dynamic, larger and more diverse representation by adding genotyped individuals with phenotypes as arise from these breeding programmes.

#### Organization of breeding programmes

Breeding programmes in perennial fruit crops may encompass different breeding themes, such as disease resistance, novel flavour and flesh colour,^2^ each requiring a separate elite population for each theme. Deploying genomic selection for each breeding theme separately would yield highest accuracy of selection as this maximizes the coincidence of key chromosome segments in training and application populations. However, this challenges the management of inbreeding due to the lower effective population size that arises from a highly related elite population. Simulation experiments on genomic selection indicated lower rates of inbreeding per generation.^70^ However, these lower rates per generation may be counteracted by the reduction in generation interval, such that the net outcome of genomic selection schemes will be an increase in inbreeding per year. Relative to trait-targeted training population, the use of a large diverse training population could reduce inbreeding, probably at the expense of prediction accuracy. Note that the high-density genotyping of breeding candidates presents an excellent opportunity to monitor genetic diversity at the genome level and to control inbreeding.

The relatively higher efficiency of genomic selection compared to conventional selection in terms of genetic gain per year was estimated considering a reduction of breeding cycle length from seven years in conventional selection to four years when using genomic selection.^23^ Apple breeders could work on further reducing generation interval to gain the full advantage of the early availability of GBV by rapid cycling.^3^ Finally, breeders could dramatically increase the number of progeny per cross and apply a higher selection intensity among juveniles based on GBV obtained from SNP profiles. The latter will incur higher costs for genotyping, so novel cost-efficient genotyping strategies, such as Genotyping by Sequencing, must be considered. More studies are needed to optimize allocation of resources for phenotyping and genotyping to maximize prediction power for Mendelian, ordinal and complex traits in fruit crops.

## Conclusion

This paper reports a substantial range in the accuracy of genomic prediction and realized selection responses for ordinal culling traits in apple. Lower accuracy and response were observed for traits with reduced or skewed phenotypic distributions and with low heritabilities. For symmetrically distributed traits with moderate or high heritability, the genomic predictions could substitute expensive field phenotyping to cull the poorest individuals with moderate intensity of selection.

## Figures and Tables

**Table 1:** Accuracy of prediction for the 10 traits within the application families, and means of these correlations over families and over different sets of traits (all: Mean_10Traits; attractiveness, fruit cropping, fruit size and per cent russet: Mean_4Traits) and mean length by family of 95% confidence intervals of these correlations.

Family name	AF1_Da66	AF2_Pi63	AF3_31Fu	AF4_31Ga	AF5_33Br	
Parent 1	‘Dalinette’	‘Pinova’	313	313	338	
Parent 2	X-6681	X-6398	‘Fuji’	‘Gala’	‘Braeburn’	
						mean
Family size	662	172	269	109	178	
Attractiveness	0.21	0.18	0.35	0.19	0.14	0.21
Fruit cropping	0.08	0.09	0.02	0.19	0.03	0.08
Fruit size	0.26	0.19	0.08	0.33	0.25	0.23
Per cent russet	0.18	0.21	0.38	0.30	−0.06	0.20
Fruit cracking	−0.09	−0.05	0.13	−0.02	0.07	0.01
Pre-harvest dropping			0.02	−0.06	−0.02	−0.02
Per cent over colour	0.31	0.22	0.50	0.46	0.36	0.37
Over colour	0.34	0.17	0.44	0.49	0.32	0.35
Ground colour	−0.03	0.12	0.09	−0.05	0.17	0.06
Type of colour	−0.06	0.00	−0.25	−0.23	−0.14	−0.14
Mean_10Traits	0.13	0.13	0.18	0.16	0.11	
Mean_4Traits	0.18	0.17	0.21	0.25	0.09	
mean length conf_interval	0.16	0.29	0.23	0.37	0.29	

**Table 2:** Marker-based relatedness (mean and standard deviation within family) and pedigree-based relatedness estimates of the five full-sib families of the application population to the training population.

	AF1_Da66	AF2_Pi63	AF3_31Fu	AF4_31Ga	AF5_33Br
Top 10	0.39 (0.040)	0.35 (0.027)	0.36 (0.037)	0.31 (0.032)	0.17 (0.028)
Top 5%	0.31 (0.030)	0.29 (0.020)	0.30 (0.030)	0.26 (0.027)	0.13 (0.019)
Top 25%	0.14 (0.014)	0.17 (0.013)	0.18 (0.014)	0.16 (0.016)	0.08 (0.008)
Pedigree-based	0.11	0.19	0.16	0.22	0.03

**Table 3:** Correlations between family-averaged relatedness estimates and accuracy of prediction for the 10 traits for different measures of relatedness.

	Top 10^a^	Top 5%^a^	Top 25%^a^	Pedigree-based
Attractiveness	0.55	0.59	0.68	0.27
Fruit cropping	0.21	0.23	0.27	0.65
Fruit size	−0.26	−0.31	−0.42	−0.01
Per cent russet	0.76	0.82	0.96^b^	0.80
Fruit cracking	−0.44	−0.37	−0.10	−0.25
Pre-harvest dropping	0.25	0.25	0.23	−0.28
Per cent over colour	−0.11	−0.06	0.17	0.12
Over colour	0.01	0.03	0.16	0.15
Ground colour	−0.58	−0.54	−0.40	−0.45
Type of colour	0.15	0.09	−0.16	−0.14

a The top 10, top 5% and top 25% relatedness of each individual of the application population to the training population was calculated as the mean of the 10, 5% or 25% highest values among the elements of **G** corresponding to the relatedness of this individual to the individuals of the training population.

b This value of correlation was significant, whereas all others were not significant.

## Appendix

Expectation of correlation between phenotypic scores (*y*) and GBV

The phenotypic scores can be modelled as

$$y=\text{X}b+\text{Z}u+\varepsilon^{P}$$

where ***y*** is a vector of phenotypic scores for a given trait, ***b*** is the vector of fixed effects (e.g. grand mean), **X** is the incidence matrix linking observations to fixed effects, **Z** is the incidence matrix linking individuals to their polygenic additive effect (= true breeding value) **u** which has a Normal distribution with $Var(u)=\text{A}\sigma_{\text{a}}^{2}$, where **A** is the pedigree-based relationship matrix and $\sigma_{\text{a}}^{2}$ is the additive genetic variance and $\varepsilon^{P}$ is a vector of residual terms identically and independently distributed with a variance $\sigma_{e}^{2}P$.

GBV ($\hat{g}$) can be considered as an estimation of **u**, and thus we have

$$\hat{g}=u+\varepsilon^{g}$$

where $\varepsilon^{g}$ is a vector of residual terms with variance $\sigma_{{\text{e}}^{G}}^{2}$.

As the GBV $\hat{g}$ are predictions derived from a model fitted to data from the training population while the phenotypic scores pertain to the application population, the residuals terms $\varepsilon^{P}$ and $\varepsilon^{g}$ are independent. It should be noted also that the usual quantity of interest is the correlation between the GBV and the polygenic additive effect/true breeding value which is

$$corr(\hat{g},u)=\frac{cov(u+\varepsilon^{g},u)}{\sqrt{var(u+\varepsilon^{g})var(u)}}=\frac{\sigma_{\text{a}}^{2}}{\sqrt{(\sigma_{\text{a}}^{2}+\sigma_{e^{G}}^{2})\sigma_{\text{a}}^{2}}}=\sqrt{\frac{\sigma_{\text{a}}^{2}}{\left(\sigma_{\text{a}}^{2}+\sigma_{e^{\text{G}}}^{2}\right)}}$$

Consequently the correlation between the GBV $\hat{g}$ and the phenotypic scores within the application FS families is

$$corr(y,\hat{g})=\frac{cov(Xb+Zu+\varepsilon^{P},u+\varepsilon^{g})}{\sqrt{var(Xb+Zu+\varepsilon^{P})var(u+\varepsilon^{g})}}=\frac{\sigma_{\text{a}}^{2}}{\sqrt{\left(\sigma_{\text{a}}^{2}+\sigma_{e^{P}}^{2})(\sigma_{\text{a}}^{2}+\sigma_{e^{G}}^{2}\right)}}$$

$$corr(y,\hat{g})=\frac{\sqrt{\sigma_{a}^{2}}\sqrt{\sigma_{a}^{2}}}{\sqrt{\left(\sigma_{a}^{2}+\sigma_{e^{P}}^{2})(\sigma_{a}^{2}+\sigma_{e^{G}}^{2}\right)}}=\sqrt{\frac{\sigma_{a}^{2}}{\left(\sigma_{a}^{2}+\sigma_{e^{P}}^{2}\right)}}\sqrt{\frac{\sigma_{a}^{2}}{\left(\sigma_{a}^{2}+\sigma_{e^{G}}^{2}\right)}}=\sqrt{h^{2}}corr(\hat{g},u)$$
