# Supplementary material for: Accuracy and responses of genomic selection on key traits in apple breeding
Source: Hortic Res. 2015 Dec 23;2:15060–. doi: 10.1038/hortres.2015.60 (PMC4688998; doi:10.1038/hortres.2015.60)
Supplement: Supplementary Data [file hortres201560-s1.docx]

### Accuracy and responses of genomic selection on key traits in apple breeding

Hélène Muranty^1*^, Michela Troggio^2^, Inès Ben Sadok^1^, Mehdi Al Rifaï^1^, Annemarie Auwerkerken^3^, Elisa Banchi^2^, Riccardo Velasco^2^, Piergiorgio Stevanato^4^, W. Eric van de Weg^5^, Mario Di Guardo^2,5^, Satish Kumar^6^, François Laurens^1^, Marco C.A.M. Bink^7*^

^1^Institut de Recherche en Horticulture et Semences UMR1345, INRA, SFR 4207 QUASAV, F-49071 Beaucouze, France

^2^Research and Innovation Center, Fondazione Edmund Mach, San Michele all’Adige, Trento, Italy

^3^Better3Fruit, Rillaar, Belgium

^4^University of Padova, Legnaro, Padova, Italy

^5^Wageningen UR Plant Breeding, Wageningen University and Research Center, Wageningen, The Netherlands

^6^The New Zealand Institute for Plant & Food Research Limited, Private Bag 1401, Havelock North 4157, New Zealand

^7^Biometris, Wageningen University and Research Center, Wageningen, The Netherlands

* corresponding authors, Helene.Muranty@angers.inra.fr or marco.bink@wur.nl

**Supplementary Data**

**Supplementary Data 1** Reference cultivars used to assess location and year effects for the phenotyping of the training population

'Akane', 'Braeburn', 'Clivia', Cox OP , 'Delicious', 'Discovery', 'Elan', 'Elstar', 'Fiesta', 'Gala', 'Gloster', 'Golden Delicious', 'Granny Smith', 'Idared', 'Ingrid Marie', 'James Grieve', 'Jonamac', 'Jonathan', 'Kent', 'McIntosh', 'Monroe', 'Mutsu', 'Pilot', 'Pinova', 'Prima', 'Priscilla', 'Red Rome', 'Rubin', 'Spartan'

**Supplementary Data 2** SNP selection process to build the 512 SNP array

The criteria for selecting the 512 SNPs were (1) the heterozygosity in the parents of the application – and training FS families, and (2) a whole genome coverage with an increased density at the ends of the chromosomes based on a first version of an integrated genetic linkage map (Jansen/Bink, personal communication). Robust performance across germplasm was not considered, as at that time this information was not yet available.

To select SNPs regularly spaced on the whole genome with an increased density at the ends of the linkage groups, each linkage group was divided in bins of equal length in its middle, bins of 1/10 of this length at the ends and bins of 4/10 of this length between the end bins and the middle bins. The number of middle bins on a linkage group was adjusted as a function of the length of the linkage group in order to limit the middle bin length to 16 cM (length adjusted to finally select 512 SNPs).

Within each bin, as many SNPs as needed were selected to obtain for each parent at least one SNP for which it was heterozygous and homozygous for the other parent of the full sib family. If the previous was not possible a SNP was chosen that was heterozygous in both parents. The SNPs were prioritized in order to select the least possible SNPs per bin, and maximum heterozygosity in the parents of the training FS families.

**Supplementary Data 3** Equations

*Variance components to estimate heritability*

To estimate narrow sense heritability, the following mixed linear model was used to estimate the variance components using only individuals of the training population:

|  | $\boldsymbol{y}=\mu\boldsymbol{1}+\mathbf{Z}\boldsymbol{u}+\varepsilon$ | **(1)** |
| --- | --- | --- |

where $\boldsymbol{y}$ is a vector of adjusted phenotypic data for a given trait, µ is an intercept and $\boldsymbol{1}$ a vector of 1, **Z** is the incidence matrix linking individuals to their polygenic additive effect **u** and $\boldsymbol{\varepsilon}$ is a vector of residual terms with a Normal distribution of variance $\sigma_{e}^{2}$. In this model, **u** has a Normal distribution with $Var\left( u \right)=\mathbf{A}\sigma_{a}^{2}$, where **A** is the pedigree-based relationship matrix (1) and $\sigma_{a}^{2}$is the additive genetic variance.

*Genomic prediction*

The model for the BayesCπ method (2) is

|  | $\boldsymbol{y}=\mu\boldsymbol{1}+\sum_{j=1}^{p} x_{j}g_{j}\delta_{j}+\boldsymbol{\varepsilon}$ | **(2)** |
| --- | --- | --- |

where $\boldsymbol{y}$ is a vector of genotypic BLUP for a given trait, of length $n_{t}$ (the size of the training population), µ is an intercept, p is the number of SNPs, $x_{j}$ is a column vector containing the genotypic data at SNP j, with elements $x_{ij}=$ 0, 1 or 2 if the genotype of individual i is AA, AB or BB, respectively, $g_{j}$ is the effect of SNP j, $\delta_{j}$ is a 0/1 indicator variable on the absence or presence of the SNP j in the model and $\boldsymbol{\varepsilon}$ is a vector of residual terms, of length $n_{t}$. The SNP effect, $g_{j}$ is a random variable assigned a prior Normal distribution, $g_{j}\sim N\left( 0, \sigma_{g}^{2} \right)$, when present in the model $\left( \delta_{j}=1 \right)$, $\delta_{j}$ is a binomial random variable with probability $\pi$, and the residual terms have a Normal distribution with variance $\sigma_{e}^{2}$. The prior for the parameter $\pi$ was uniform.

The GBV in the application population, $\hat{\boldsymbol{g}}$**,** were obtained by

|  | $\hat{\boldsymbol{g}}=\hat{\mu}\boldsymbol{1}+\sum_{j=1}^{p} x_{j}\hat{g}_{j}\hat{\delta}_{j}$ | **(3)** |
| --- | --- | --- |

where $\hat{\mu}$, $\hat{g}_{j}$ and $\hat{\delta}_{j}$ are the calculated estimates for the intercept, SNP effects and indicator variable, respectively.

To obtain an initial value for $\sigma_{g}^{2}$ and $\sigma_{e}^{2}$, the data were first analysed using the same model as in equation (1) but with $\boldsymbol{y}$ being the vector of genotypic BLUP for a given trait.

The initial value for $\sigma_{g}^{2}$ was then computed as

|  | $\frac{\sigma_{a}^{2}}{2\sum_{j=1}^{p} f_{j}\left( 1-f_{j} \right)}$ | **(4)** |
| --- | --- | --- |

where $f_{j}$ is the allelic frequency at SNP *j* in the training population.

1. Lynch M, Walsh B. Genetics and analysis of quantative traits. Sinauer Associates Incorporated; Sunderland; USA; 1997. xvi + 980 pp. p.

2. Habier D, Fernando RL, Kizilkaya K, Garrick DJ. Extension of the Bayesian alphabet for genomic selection. BMC Bioinformatics. 2011;12(1):186.
