# Supplementary material for: Accuracy and responses of genomic selection on key traits in apple breeding
Source: Hortic Res. 2015 Dec 23;2:15060–. doi: 10.1038/hortres.2015.60 (PMC4688998; doi:10.1038/hortres.2015.60)
Supplement: Supplementary Figure Legends [file hortres201560-s2.docx]

**Accuracy and responses of genomic selection on key traits in apple breeding**

Hélène Muranty^1*^, Michela Troggio^2^, Inès Ben Sadok^1^, Mehdi Al Rifaï^1^, Annemarie Auwerkerken^3^, Elisa Banchi^2^, Riccardo Velasco^2^, Piergiorgio Stevanato^4^, W. Eric van de Weg^5^, Mario Di Guardo^2,5^, Satish Kumar^6^, François Laurens^1^, Marco C.A.M. Bink^7*^

^1^Institut de Recherche en Horticulture et Semences UMR1345, INRA, SFR 4207 QUASAV, F-49071 Beaucouze, France

^2^Research and Innovation Center, Fondazione Edmund Mach, San Michele all’Adige, Trento, Italy

^3^Better3Fruit, Rillaar, Belgium

^4^University of Padova, Legnaro, Padova, Italy

^5^Wageningen UR Plant Breeding, Wageningen University and Research Center, Wageningen, The Netherlands

^6^The New Zealand Institute for Plant & Food Research Limited, Private Bag 1401, Havelock North 4157, New Zealand

^7^Biometris, Wageningen University and Research Center, Wageningen, The Netherlands

* corresponding authors, Helene.Muranty@angers.inra.fr or marco.bink@wur.nl

**Supplementary Figure Legends**

**Supplementary Fig. S1** Pedigree of the training population and the application families, visualized with Pedimap software (1).

**Supplementary Fig. S2** Positions of the selected 512 SNPs on the genetic map and their distribution into the non-scored, robust and non-robust classes.

**Supplementary Fig. S3** Normal quantile-quantile plots of residual terms of the genomic prediction model in the training population.

**Supplementary Fig. S4** Marker-based relatedness at the individual level, between individuals of the training population and individuals of the application population, sorted by full-sib families. The colour scale on the right indicates the level of relatedness, from the lowest (-0.31, dark blue) to the highest (0.59, dark red).

**Supplementary Fig. S5** **A** Distribution of linkage disequilibrium (LD), measured as *r*^2^, among adjacent marker pairs in the training population. **B** Average LD, measured as *r*^2^, for pairs of markers in increments of 0.01 cM, according to the genetic distance between the markers. The red line was obtained by fitting a cubic smoothing spline to the data.

1. Voorrips RE, Bink MCAM, Weg WE van de. Pedimap: Software for the Visualization of Genetic and Phenotypic Data in Pedigrees. J Hered. 2012;103(6):903‑7.
