# Supplementary material for: Accuracy and responses of genomic selection on key traits in apple breeding
Source: Hortic Res. 2015 Dec 23;2:15060–. doi: 10.1038/hortres.2015.60 (PMC4688998; doi:10.1038/hortres.2015.60)
Supplement: Supplementary Tables [file hortres201560-s3.docx]

### Accuracy and responses of genomic selection on key traits in apple breeding

Hélène Muranty^1*^, Michela Troggio^2^, Inès Ben Sadok^1^, Mehdi Al Rifaï^1^, Annemarie Auwerkerken^3^, Elisa Banchi^2^, Riccardo Velasco^2^, Piergiorgio Stevanato^4^, W. Eric van de Weg^5^, Mario Di Guardo^2,5^, Satish Kumar^6^, François Laurens^1^, Marco C.A.M. Bink^7*^

^1^Institut de Recherche en Horticulture et Semences UMR1345, INRA, SFR 4207 QUASAV, F-49071 Beaucouze, France

^2^Research and Innovation Center, Fondazione Edmund Mach, San Michele all’Adige, Trento, Italy

^3^Better3Fruit, Rillaar, Belgium

^4^University of Padova, Legnaro, Padova, Italy

^5^Wageningen UR Plant Breeding, Wageningen University and Research Center, Wageningen, The Netherlands

^6^The New Zealand Institute for Plant & Food Research Limited, Private Bag 1401, Havelock North 4157, New Zealand

^7^Biometris, Wageningen University and Research Center, Wageningen, The Netherlands

* corresponding authors, Helene.Muranty@angers.inra.fr or marco.bink@wur.nl

**Supplementary Tables**

Supplementary Table S1 Specification on which FS families were involved in subsequent steps. Parents involved in both the training and application populations are in bold.

| Part of the material | FS families | Number of individuals | Step 1:  genotype imputation | Step 2:  model training | Step 3:  model application |
| --- | --- | --- | --- | --- | --- |
| Additional reference | 'Telamon' × 'Braeburn', 'Jonathan' × 'Prima' | 187 | X |  |  |
| Training population | 'Discovery' × 'Prima', **'Fuji'** × **'Gala'**, **'Fuji'** × **'Pinova'**, **'Gala'** × 'Cripps Pink', **'Gala'** × **'Pinova'**, **'Pinova'** × 'Reanda', RedWinterX3177 × 'Galarina', 'Rewena' × 'Pirol', 'Rubinette' × X‑3305, X‑3263 × X‑3259, X‑3305 × X‑3259, X‑3318 × 'Galarina', X‑3318 × X‑3263, X‑3318 × X‑6564, **X‑6398** × X‑6683, X‑6417 × X‑6564, X‑6679 × 'Dorianne', X‑6679 × X‑6417, X‑6679 × X‑6808, X‑6683 × **X‑6681** | 977 | X | X |  |
| Application population | 'Dalinette' × **X-6681**, **'Pinova'** × **X-6398**, 313 × **'Fuji'**, 313 × **'Gala'**, 338 × 'Braeburn' | 1390 | X |  | X |

Supplementary Table S2 Descriptors used to score the training and application populations

|  | 1 | 2 | 3 | 4 | 5 |
| --- | --- | --- | --- | --- | --- |
| Attractiveness | not at all attractive | not attractive | just OK | nice | very nice |
| Fruit cropping | very low  (< 10fruits) | low | good | high | very high |
| Fruit size | very small | small | middle  (65-80mm) | big | very (too) big |
| Percent of russet | no | some spots (<5%) | significant  (5-20%) | high  (20-50%) | very important  (>50%) |
| Fruit cracking | no | some spots | significant | high | very high |
| Pre-harvest dropping | very low  (<1%) | low | middle | high | very high |
| Percent over-colour | very low  (< 1%) | low  (<25%) | middle  (25-50%) | high  (50-75%) | very high  (>75%) |
| Over-colour | none |  |  |  | deep red |
| Ground colour | green |  |  |  | yellow |
| Type of colour | strip | strip-blush | blush-strip | blush |  |
| Attractiveness of colour | not at all attractive |  |  |  | nice |

Supplementary Table S3 Realized selection differential expressed as the mean phenotypic difference between the best individuals (highest GEBV) and the worst individuals (lowest GEBV). The number of best and worst individuals varied according to family size and has been indicated as number selected. In bold, significant differences (P < 5%).

|  | AF1_Da66 | AF2_Pi63 | AF3_31Fu | AF4_31Ga | AF5_33Br |
| --- | --- | --- | --- | --- | --- |
| Number selected^a^ | 50 | 13 | 20 | 8 | 13 |
| Attractiveness | **0.70** | **0.62** | **0.95** | **1.38** | 0.23 |
| Fruit cropping | 0.32 | 0.38 | 0.10 | **1.13** | 0.23 |
| Fruit size | **0.60** | 0.38 | 0.30 | **0.75** | **0.54** |
| Percent russet | 0.08 | **0.46** | **0.13** | **1.13** | -0.31 |
| Fruit cracking | -0.08 | 0.00 | 0.30 | -0.13 | 0.31 |
| Pre-harvest dropping |  |  | 0.05 | 0.13 | 0.00 |
| Percent over-colour | **0.58** | **1.00** | **1.60** | **2.63** | **1.15** |
| Over-colour | **0.88** | 0.77 | **1.45** | **2.38** | **1.00** |
| Ground colour | -0.20 | 0.31 | 0.40 | -0.13 | **0.85** |
| Type of colour | -0.08 | 0.08 | **-0.90** | -1.13 | -0.77 |

^a^ the number of individuals selected corresponds to 7.5% of the size of each FS family (see Table 1)

Supplementary Table S4 Directional realized selection differential expressed as the difference between the best individuals and the family mean, the number of best individuals varying according to family size and indicated as number selected. In bold, significant differences (P < 5%). Ground colour and type of colour were not included in this table as breeders did not indicate a preferred selection direction.

|  | selection direction | AF1_Da66 | AF2_Pi63 | AF3_31Fu | AF4_31Ga | AF5_33Br |
| --- | --- | --- | --- | --- | --- | --- |
| Number selected^a^ |  | 50 | 13 | 20 | 8 | 13 |
| Attractiveness | **+** | **0.35** | 0.19 | **0.51** | 0.27 | -0.11 |
| Fruit cropping | + | 0.18 | 0.19 | 0.21 | **0.69** | 0.18 |
| Fruit size | **+** | **0.38** | **0.47** | 0.03 | 0.16 | 0.19 |
| Percent russet | **-** | **-**0.09 | -**0.42** | -**0.69** | -**1.03** | 0.02 |
| Fruit cracking | - | 0.09 | -0.02 | **-0.13** | -0.09 | **-0.27** |
| Pre-harvest dropping | - |  |  | -0.02 | **-0.14** | -0.04 |
| Percent over-colour | **+** | **0.41** | **0.52** | **0.75** | **0.88** | **0.75** |
| Over-colour | **+** | **0.51** | 0.37 | **0.55** | 0.62 | 0.60 |

^a^ the number of individuals selected corresponds to 7.5% of the size of each FS family (see Table 1)
