# Supplementary figures and images for: Accuracy and responses of genomic selection on key traits in apple breeding
Source: Hortic Res. 2015 Dec 23;2:15060–. doi: 10.1038/hortres.2015.60 (PMC4688998; doi:10.1038/hortres.2015.60)

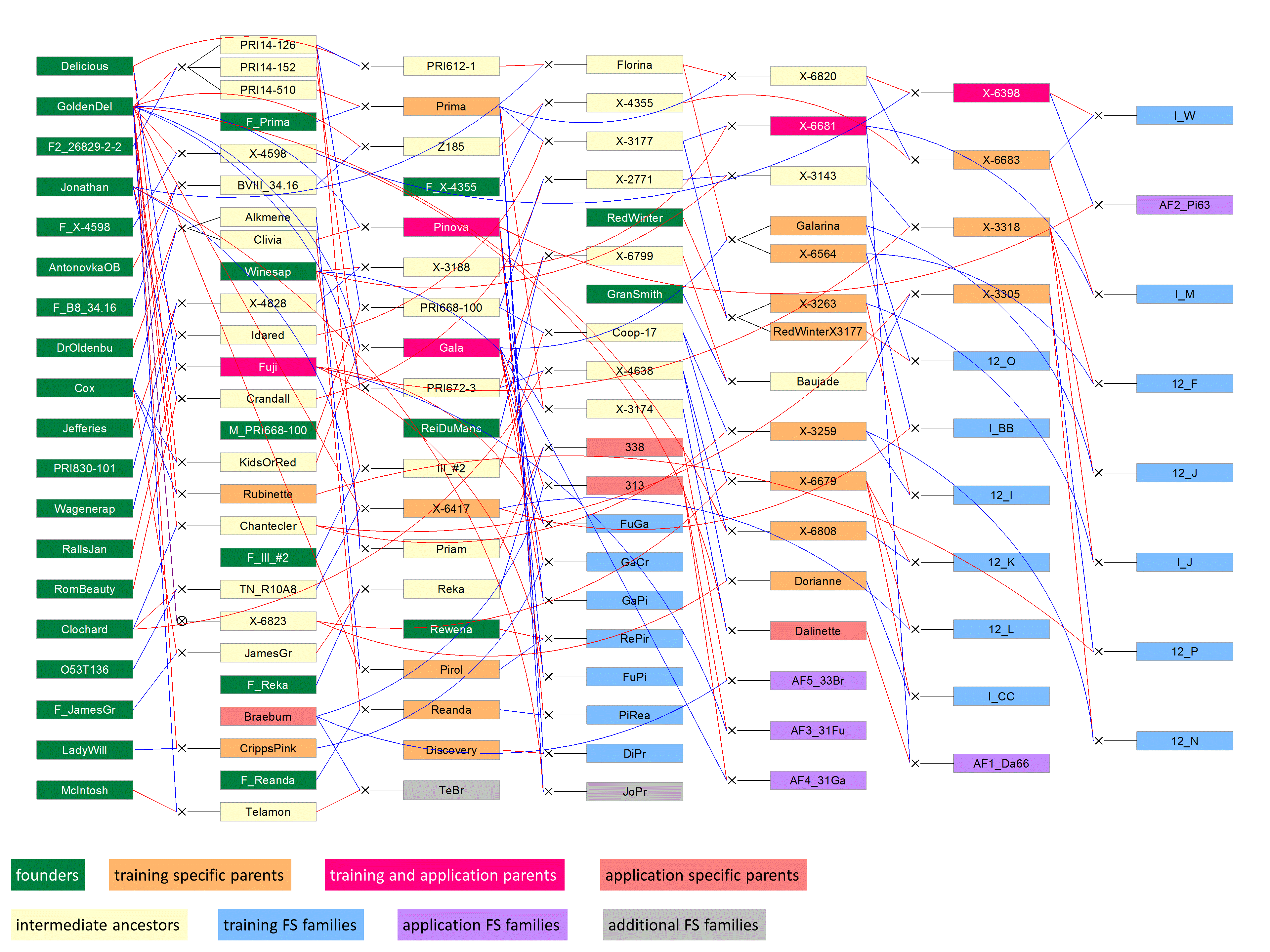

Supplement: Supplementary Figure S1 [file hortres201560-s4.gif]

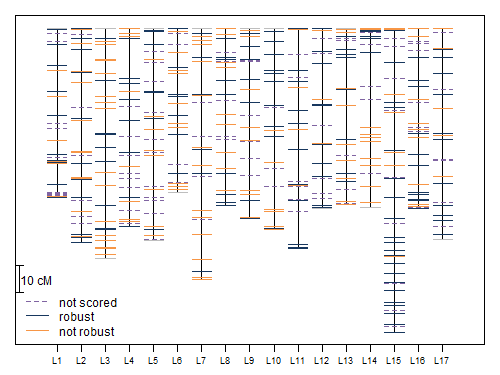

Supplement: Supplementary Figure S2 [file hortres201560-s5.gif]

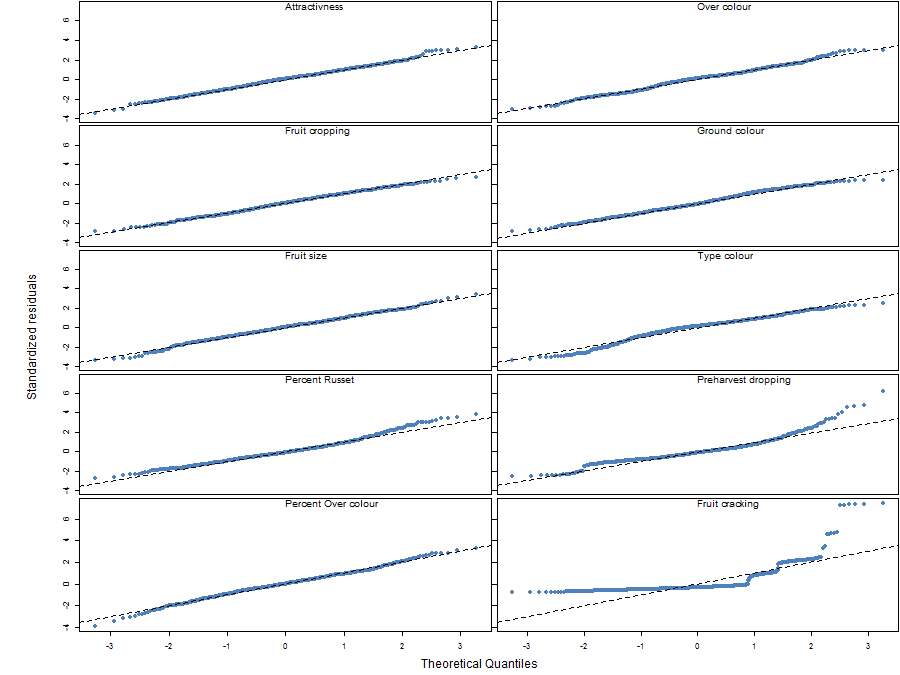

Supplement: Supplementary Figure S3 [file hortres201560-s6.gif]

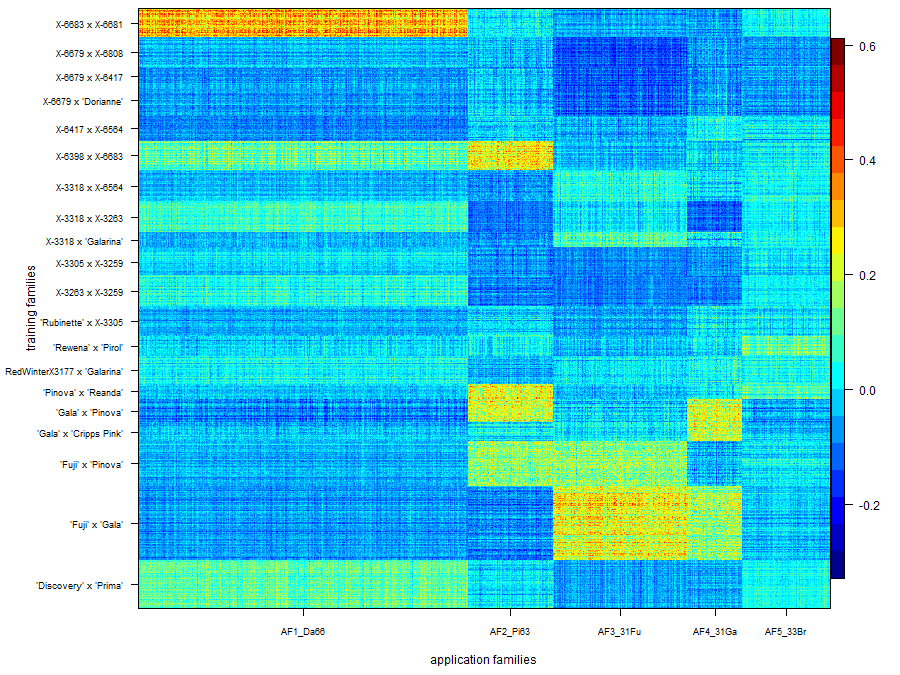

Supplement: Supplementary Figure S4 [file hortres201560-s7.gif]

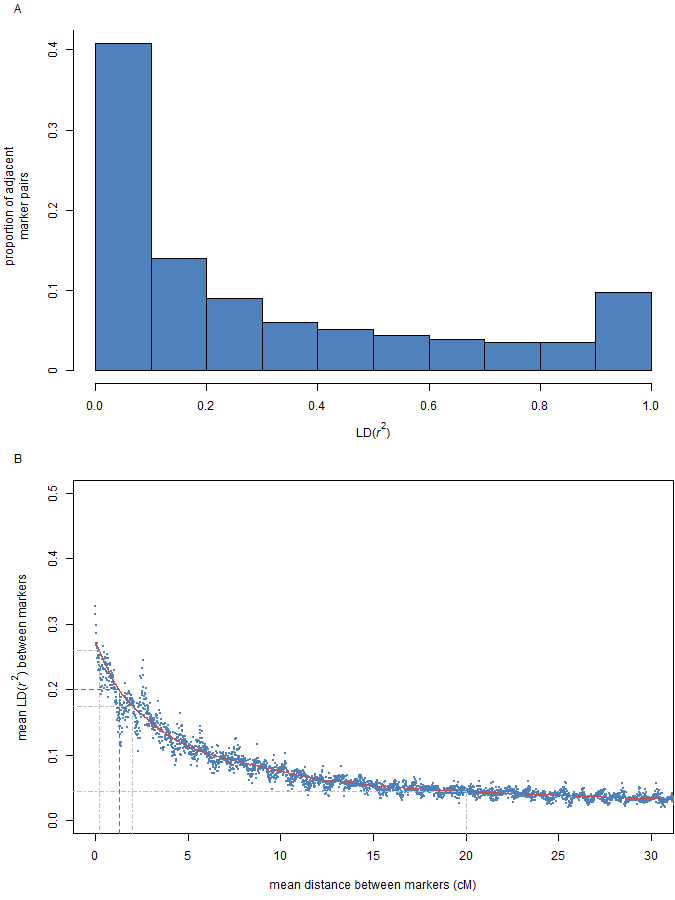

Supplement: Supplementary Figure S5 [file hortres201560-s8.gif]
